# Efficacy, safety, and tolerability of Epalrestat compared to Methylcobalamine in patients with diabetic neuropathy

**DOI:** 10.4103/0973-3930.50712

**Published:** 2009

**Authors:** Manish Maladkar, Girish Rajadhyaksha, N. Venkataswamy, R. S. Hariharan, Sathis R. Lohati

**Affiliations:** Aristo Pharmaceuticals P Ltd Mumbai-53, India; 1 LTMMC and LTMG Hospital, Sion West, Mumbai-22, India; 2 Shreya hospital Bangaluru-60, India; 3 Diabetes and Heart care Hospital P Ltd, Chennai-61, India; 4 MR Medical College Gulbarga-585104, India

**Keywords:** Diabetic neuropathy, Epalrestat, Methylcobalamin

## Abstract

**OBJECTIVE:**

To compare the efficacy, safety, and tolerability of Epalrestat with Methylcobalamine in patients with diabetic neuropathy.

**MATERIALS AND METHODS:**

This prospective, open-labeled, comparative, and multicentric study was conducted on an outpatient basis by qualified investigators at four different centers. A total of 242 patients with diabetic neuropathy were enrolled in the study. The enrollment of patients was as per inclusion/exclusion criteria. After obtaining their informed consent, they were individually interviewed, examined, investigated, and treated as per the study protocol. Each patient was administered with one tablet containing Epalrestat 50 mg thrice daily or Methylcobalamine 500 µg thrice daily for 12 weeks. They were followed up at the end of weeks 4, 8, and 12 for their examination. Therapeutic success was assessed in terms of clinical symptoms, physical examinations, and electrophysiological assessments.

**RESULTS:**

A significant improvement in all the efficacy parameters was observed with Epalrestat treatment compared to Methylcobalamin treatment. The efficacy parameters assessed were loss of sensation, burning sensation, numbness, muscle cramps, spontaneous pain, weakness, mean score of isometric muscle strength, tendon reflexes, and sensation. Epalrestat treatment is associated with very few adverse events. The tolerability to Epalrestat treatment was also reported to be excellent in the majority of the study population compared to tolerability to Methylcobalamine. Patients as well as physicians reported that the Epalrestat treatment is superior in efficacy and safety parameters compared to Methylcobalamin.

**CONCLUSION:**

The present study concludes that Epalrestat has better efficacy and safety profile than Methylcobalamine in the treatment of diabetic neuropathy.

## Introduction

Peripheral neuropathy is one of the most common and disabling long-term complications of diabetes mellitus. Diabetic neuropathy is the condition, either clinically evident or subclinical, that occurs in diabetes mellitus cases in the absence of other causes of peripheral neuropathy. Diabetic neuropathy remains the least understood and most difficult-to-treat diabetic complication.

At the time of diagnosis of diabetes, 12% of the patients have been found to have neuropathy, the prevalence increasing to 50% by ten years after diagnosis. One US-based survey shows 60–70% of diabetics have some kind of nervous system damage. Attempts to prevent or avert the course of diabetic neuropathy have been made using a variety of drugs. The symptoms of diabetic neuropathy often go unnoticed at first, and sometimes for a long time. Numbness, pain, or tingling in the feet or legs which are the common symptoms of disease may appear after several years. Nerve damage in diabetic neuropathy is a continuous process and over a period of years, it mainly affects the digestive and reproductive systems.

Apart from maintaining blood glucose levels within the acceptable range, the goal of treating diabetic neuropathy is to relieve discomfort and to prevent further nerve damage. Analgesics, low doses of antidepressants, and some anticonvulsant medications may be prescribed for the relief of pain, burning, or tingling. Some patients may find that walking regularly, taking warm baths, or using elastic stockings may help to relieve leg pain.

Epalrestat is a novel aldose reductase inhibitor which has been proven to have beneficial effects in diabetic neuropathy in many controlled clinical trials.(3–7) It has been suggested that accumulation of sorbitol in certain cells occurring only in conditions of hyperglycemia and resulting in a hyperosmotic effect, may be involved in the pathogenesis of some diabetic complications. Epalrestat inhibits sorbitol production and prevents further complications. This agent has no influence on blood glucose concentrations.

Methylcobalamine, a form of vitamin B12, has been used in diabetic neuropathy for many years and has shown benefits in other neuromuscular diseases as well. Unlike regular B12 (cyanocobalamine), Methylcobalamine is active in the spinal fluid. Due to this property, it is able to help heal the damaged nerve cells and restore normal functions. In a double-blind study, the active group treated with Methylcobalamine showed statistical improvement in somatic and autonomic symptoms with the regression of signs of diabetic neuropathy. The drug was easily tolerated by patients and no side effects were encountered.(2)

The objective of the present study was to compare the efficacy, safety, and tolerability of Epalrestat with Methylcobalamine in patients with diabetic neuropathy.

## Materials and Methods

This prospective, randomized, single-blind, comparative study was conducted on an outpatient basis by qualified investigators at four different sites.

Patients with diabetic neuropathy who were attending the clinic regularly were considered for the study. The purpose and nature of the study was explained to the patients by an investigator. Those patients who expressed their willingness to participate were screened for their suitability for the study as per the inclusion and exclusion criteria after obtaining their informed consent. Only those patients who fulfilled the requirements were enrolled in the study.

Inclusion criteria for enrollment were: 1) Age ≥ 18 years, 2) patients diagnosed with type 2 diabetes mellitus treated with altered diet or oral hypoglycemic agents, with evidence of clinical, symptomatic, symmetric, distal polyneuropathy, and 3) Patients ready to give informed consent.

The following patients were excluded from the study: 1) Those with any neuropathy other than diabetic neuropathy, 2) Those with cauda equina or spinal cord disease and/or diseases related to the peripheral nerves, 3) Those on antidepressants, anticonvulsants, opiates, mexilitine, capsaicin, neuroleptics, vitamin B6 compounds, aldose deductase inhibitors, and antioxidants, 4) Those with peripheral vascular disease and other severe diseases, 5) Those with impaired hepatic or renal function, and 6) Those with hypersensitivity to the study medication, and 7) Those suffering from any physiological or pathological condition as these could alter the results of the study.

The study patients were screened for their demographic profiles which included age, sex, and weight. Normal investigations were carried out which included an electrocardiogram, an X-ray of the chest, and urine analysis. The patients were also subjected to normal biochemical and hematological analyses.

The total number of patients selected for the study was 242. The enrolled patient population was divided into two groups: each patient of the first group was administered with one tablet containing Epalrestat 50 mg thrice daily whereas each patient of the second group was administered Methylcobalamine 500 μg thrice daily for 12 weeks. They were followed up at the end of weeks 4, 8, and 12 for their examination. Clinical examination was done by the attending physicians and the signs and symptoms were recorded. All patients were asked not to take any other medication without the permission of the treating physicians.

Diabetic neuropathy was assessed in terms of clinical symptoms and physical examinations as follows:

- Symptoms: Loss of sensation, burning sensation, numbness, muscle cramps, spontaneous pain, and weakness.
- Signs: Reflexes, vibration sense, lower motor neuron weakness.
- Assessment: The global assessment was carried out at the end of week 12 by the investigator.

In addition to the patients' responses, investigators' opinions were also recorded. Global efficacy and tolerability of the treatment were judged on a four-point scale by the investigators as being excellent, good, fair, and poor.

As the reporting and recording adverse events are of utmost importance, the study population was kept under observation during the entire study period for any adverse event. In addition to the monitoring records, patients were asked to keep a record of, and report on their own, any unwanted effects experienced during the study period.

The data from different centers were pooled, tabulated, and analyzed using the appropriate parametric and nonparametric tests.

## Results

A total of 242 patients suffering from diabetic neuropathy were enrolled in the study after screening the patient population at four different centers. Out of 242, 230 patients completed the study; dropouts from the study were because of individual patients' noncompliance.

### Demographic profiles

The demographic profiles of the patients enrolled in the study as per the inclusion-exclusion criteria are shown in Table 1. The enrolled patients were in the age group of 30 to 64 years with mean ages of 50.45 years for the Epalrestat group and 53.05 years for the Methylcobalamine group. Other parameters are also listed in Table 1.

**Table 1:** Demographical characteristics of the study population

Parameters	Epalrestat Group	Methylcobalamine Group
*n*	120	122
Age* (Yrs)		
Mean	50.45	53.05
SD	9.27	9.79
Range	32–58 yrs	30–64 yrs
Weight* (Kg)		
Mean	58.16	60.25
SD	10.13	10.95
Range	52–75 kg	51–78 kg
Sex# (%)		
Male	76 (63.3)	78 (63.9)
Female	44 (36.7)	44 (36.1)

* ANOVA,

# By Chi-square test *P* > 0.05 Not Significant

### Physical and Laboratory examination

All parameters including pulse rate, blood pressure, and respiratory rate were within normal limits at baseline. Laboratory investigations for RBC, WBC, ESR, SGOT, SGPT, Na, K, and bilirubin were within normal limits.

### Efficacy Parameters

#### Loss of sensation

Loss of sensation is one of the major symptoms of diabetic neuropathy. In both the study groups, almost all the cases had similar loss of sensation at baseline with no significant difference between the groups. After treatment at the end of the 4^th^ week, the proportion of cases with loss of sensation had significantly fallen in both the groups from the baseline. At the end of the 12^th^ week of treatment, only 15.7% of the cases from the Epalrestat group remained with loss of sensation whereas the unimproved population was 28.7% in the Methylcobalamine group, which was significantly greater than in the Epalrestat group [Table 2].

**Table 2:** Effect on loss of sensation

Duration in Weeks	Study population with sensory loss			
	
	Epalrestat (*N* = 115)		Methylcobalamin (*N* = 115)	
		
	*n*	%	*n*	%
Baseline	115	100.0	115	100.0
4	*83	72.2	*94	81.7
8	*38	33.0	@*61	53.0
12	*18	15.7	@*33	28.7

By Chi-Square Test

* *P* < 0.05 Significant,

@ Between Groups

### Burning sensation

Burning feet are a common complaint of diabetic neuropathy and this complaint becomes prominent over the age of 50 years. Table 3 reveals that almost all the cases in both the groups had a burning sensation at baseline with no significant difference within the groups. Both the treatments showed significant improvement during the entire treatment period. At the end of treatment, *i.e*., 12 weeks, there was significant improvement in the Epalrestat group (25.2%) compared to the Methylcobalamine group (11.3%).

**Table 3:** Effect on burning sensation

Duration in Weeks	Study population with burning			
	
	Epalrestat (*N* = 115)		Methylcobalamin (*N* = 115)	
		
	*n*	%	*n*	%
Baseline	115	100.0	115	100.0
4	*77	67.0	*84	73.0
8	*26	22.6	@*57	49.6
12	*13	11.3	@*29	25.2

By Chi-Square Test,

* *P* < 0.05 Significant,

@ Between Groups

### Numbness

The majority (85.2–86.1%) of the cases in both groups had numbness at baseline. After the treatment at the end of the 4^th^ week, the proportion of cases with numbness had significantly fallen from baseline in both the groups. Some (43.5%) of the cases from the Epalrestat group did not have numbness which was a significant improvement as compared to 28.7% of the cases in the Methylcobalamine group. At the end of the 12^th^ week, 27.0% of the cases from the Methylcobalamine group had numbness which was significantly more than the 13.0% in the Epalrestat group [Table 4] shows the difference between the two groups—the efficacy of Epalrestat in improving numbness is clearly better than Methylcobalamine.

**Table 4:** Effect on numbness

Duration in Weeks	Study population with numbness			
	
	Epalrestat (*N* = 115)		Methylcobalamin (*N* = 115)	
		
	*n*	%	*n*	%
Baseline	98	85.2	99	86.1
4	*65	56.5	@*82	71.3
8	*34	29.6	@*55	47.8
12	*15	13.0	@*31	27.0

By Chi-Square Test,

* *P* < 0.05 Significant,

@ Between Groups

### Muscle cramps

In the present study, 76.5–78.2% of the cases in both the groups had similar complaints of muscle cramps at baseline with no significant difference between the groups. During the treatment, the proportion of cases with muscle cramps in both the groups had significantly fallen from baseline. Significant improvement was observed in the Epalrestat group compared to the Methylcobalamine group at the end of the 8^th^ and 12^th^ weeks of therapy [Table 5].

**Table 5:** Effects on muscle cramps

Duration in Weeks	Study population with muscle cramps			
	
	Epalrestat (*N* = 115)		Methylcobalamin (*N* = 115)	
		
	*n*	%	*n*	%
Baseline	88	76.5	90	78.3
4	*62	53.9	*74	64.3
8	*32	27.8	@*51	44.3
12	*13	11.3	@*31	27.0

By Chi-Square test,

* *P* < 0.05 Significant,

@ Between Groups

### Spontaneous pain

More than half (59.1–60.9%) of the recruited population in both groups had spontaneous pain at baseline. The symptom of spontaneous pain was found to fall significantly with both the treatments. The Epalrestat regimen showed a significant benefit over Methylcobalamine treatment at the end of week 12 in providing relief from spontaneous pain [Table 6].

**Table 6:** Effects on spontaneous pain

Duration in Weeks	Study population with spontaneous pain			
	
	Epalrestat (*N* = 115)		Methylcobalamin (*N* = 115)	
		
	*n*	%	*n*	%
Baseline	70	60.9	68	59.1
4	*46	40.0	57	49.6
8	*23	20.0	*35	30.4
12	*09	07.8	@*22	19.1

By Chi-Square Test,

* *P* < 0.05 Significant,

@ Between Groups

### Mean score of isometric muscle strength

Score were given from 0 (complete paralysis) to 5 (normal) for each patient. The movements assessed for giving these scores were wrist flexion, finger flexion and extension, finger and thumb spread, ankle dorsiflexion and plantar flexion, and toe flexion and extension. The scores were added for each limb and divided by the number of movements assessed to give a single upper or lower limb score. Mean scores of isometric muscle strength were 2.95 and 2.87, with no significant difference in both the groups at baseline. These scores improved during the study. At the end of the 12^th^ week, increases were 48.5% in the Epalrestat group (Score: 4.38) and 37.8% in the Methylcobalamine group (Score: 3.96) [Table 7].

**Table 7:** Mean score of isometric muscle strength

Duration in Weeks	Mean score of Isometric muscle strength ($\bar{\text{X}}$ ± SD)	
	
	Epalrestat	Methylcobalamin
Baseline	2.95 + 1.30	2.87 + 1.51
4	*3.91 + 1.49	@*3.26 + 1.56
8	*4.02 + 1.64	@*3.70 + 1.42
12	*4.38 + 1.56	@*3.96 + 1.47

By ANOVA,

* *P* < 0.05 Significant,

@ Between Groups

### Tendon reflexes

Tendon reflexes were measured on a three-point scale: 0 = absent, 1 = present with reinforcement, and 2 = normal, for triceps, biceps, and bronchioradialis in the upper limb and quadriceps femoris and gastrocnemius in the lower limb. The average single scores were then calculated for the upper and the lower limbs. Table 8 reveals that none of the patients had any normal tendon reflexes at baseline. After the treatment at the end of the 4^th^ week, significantly more patients (27.0%) from the Epalrestat group had normal tendon reflexes compared to 13.9% of the Methylcobalamine group. Similarly, at the end of the 12^th^ week, significantly more patients (53.9%) from the Epalrestat group had normal tendon reflexes compared to 33.9% from the Methylcobalamine group [Table 8].

**Table 8:** Effects on Tendon reflex

Duration in weeks	Epalrestat (*N* = 115)			Methylcobalamin (*N* = 115)		
		
	Absent *n* (%)	Present with reinforcement *n* (%)	Normal *n* (%)	Absent *n* (%)	Present with reinforcement *n* (%)	Normal *n* (%)
Baseline	39 (33.9)	76 (66.1)	0 (0)	43 (37.4)	72 (62.6)	0 (0)
4	29 (25.2)	55 (47.8)	@*31 (27.0)	38 (33.1)	61 (53.0)	*16 (13.9)
8	19 (16.5)	45 (39.1)	@*51 (44.4)	30 (26.1)	55 (47.8)	*30 (26.1)
12	13 (11.3)	40 (34.8)	@*62 (53.9)	22 (19.1)	54 (47.0)	*39 (33.9)

By Chi-Square Test,

* *P* < 0.05 Significant,

@ Between Groups

### Sensation

Sensation was measured on a three-point scale (0 = absent, 1 = present with reinforcement, and 2 = normal) for the following parameters in the hand and the foot: vibration perception was examined with a 128 Hz tuning fork, pain sensation with a disposable pin, light touch with a monofilament, joint position on the fingers and toes, and thermal sensation with cold metal. All cases from both the groups showed absent to reduced sensation at baseline. After the treatment at the end of the 4^th^ week, significantly more (19.1%) number of cases from the Epalrestat group had normal sensation as compared to 8.7% in the Methylcobalamine group. At the end of the 12^th^ week, 57.4% of the cases from the Epalrestat group had normal sensation, a number which was significantly greater than the 34.8% from the Methylcobalamine group [Table 9].

**Table 9:** Effect on sensation

Duration in weeks	Epalrestat (*N* = 115)			Methylcobalamin (*N* = 115)		
		
	Absent *n* (%)	Reduced *n* (%)	Normal *n* (%)	Absent *n* (%)	Reduced *n* (%)	Normal *n* (%)
Baseline	58 (50.4)	57 (49.6)	0 (0)	59 (51.3)	56 (48.7)	0 (0)
4	34 (29.6)	59 (51.3)	@*22 (19.1)	44 (38.3)	61 (53.0)	*10 (08.7)
8	24 (20.9)	51 (44.3)	@*40 (34.8)	33 (28.7)	61 (53.0)	*21 (18.3)
12	15 (13.0)	34 (29.6)	@*66 (57.4)	22 (19.1)	53 (46.1)	*40 (34.8)

By Chi - Square Test,

* *P* < 0.05 Significant,

@ Between Groups

### Laboratory investigations

Laboratory investigations including RBC, WBC, ESR, SGOT, SGPT, Na, K, and bilirubin did not show any significant change in either group.

### Overall Global Efficacy by the Investigator

**Figure 1:**
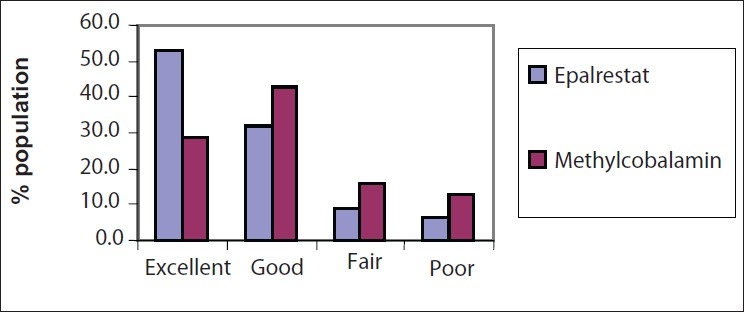
Overall global efficacy by the investigator

According to the investigators, 53.0% of the cases in the Epalrestat group showed excellent response to the treatment, a number that was significantly more than the 28.7% in the Methylcobalamine group [Figure 1].

### Overall global tolerability by the Investigators

**Figure 2:**
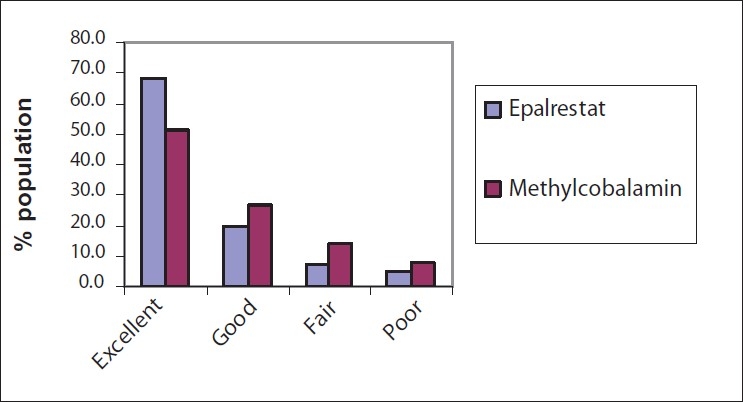
Overall global tolerability by the investigator

Results reveals that 67.8% of the cases showed excellent tolerance of treatment in the Epalrestat group which was significantly more than the 51.3% in the Methylcobalamine group [Figure 2].

### Overall global efficacy by the patients

**Figure 3:**
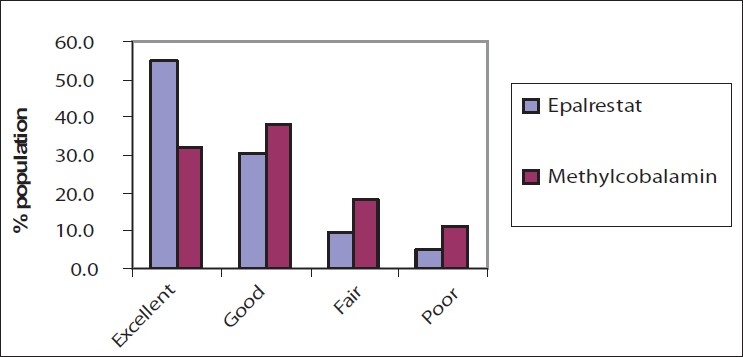
Overall global efficacy by the patients

More than half (54.8%) of the patients experienced an excellent response with Epalrestat, which was significantly greater than the 32.2% with Methylcobalamine treatment. Both treatments showed good effects on some (30.4–38.2%) of the cases [Figure 3].

### Overall global tolerability by the patients

**Figure 4:**
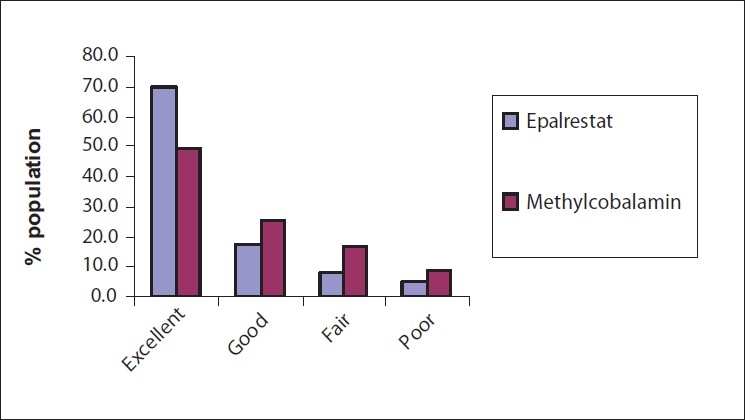
Overall global tolerability by the patients

In the Epalrestat group, 69.6% of the patients showed excellent tolerance of treatment, a number that was significantly greater than the 49.6% in the Methylcobalamine group. Some (17.4–25.2%) of the cases had a good tolerance to the treatment in both the groups [Figure 4].

### Adverse events

**Figure 5:**
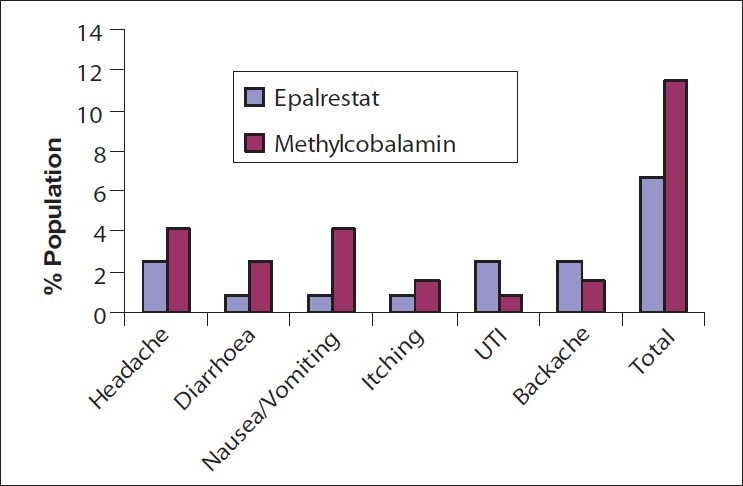
Adverse events

None of the patients experienced any serious adverse events during the study period. In the Epalrestat group, 6.7% of the patients reported various side effects whereas 11.5% of patients in the Methylcobalamine group reported side effects. The common side effect was headache; the details of adverse events have been shown in Figure 5. Also, there were no significant changes in the biochemical and hematological parameters before and after the study.

## Discussion

Diabetic neuropathy develops in the majority of poorly controlled, diabetic patients as a late complication of diabetes. Treatment is of utmost importance as 60–70% of this population may progress to suffer from serious life-threatening complications.

The rationale followed for the treatment of diabetic neuropathy includes symptomatic relief of complications besides good glycemic control. Methylcobalamine has a long history as a nerve rejuvenator and it has been used in the treatment of neuropathy for a long time. Epalrestat is a relatively newer addition in this category that has gained the acceptance of the medical community as an effective treatment option for diabetic neuropathy. The efficacy and tolerability of both these drugs were compared in patients of diabetic neuropathy with similar demographic profiles. The symptomatic relief was measured in terms of regained or altered sensation, reduction in pain and cramps, numbness, and general weakness. From the results of the study, we can conclude that Epalrestat is better than Methylcobalamine in every measured efficacy parameter. These parameters represent most of the encountered symptoms in diabetic neuropathy, thus, an improvement in these parameters is the indication of overall symptomatic improvement in the disease condition.

Ease of movement and skeletal muscle reflexes give a better idea about recovery from fatigue and weakness caused by nerve dysfunction. The mean score of isometric muscle strength is a good indicator of easy movements. This score is counted by analyzing muscle movements of different parts of the body, particularly, the extremities which are most affected in diabetic neuropathy. Statistically significant higher mean scores of isometric muscle strength for Epalrestat compared to Methylcobalamine indicate that Epalrestat has an advantage in relieving muscle weakness. The next parameter measured was tendon reflex, which shows the reflex ability of nervous tissue and attached muscle to external stimuli. The recovery of normal reflex mechanisms was greater in patients treated with Epalrestat compared to Methylcobalamine.

Most of the investigators who participated in the study felt that Epalrestat scored over Methylcobalamine in terms of safety and efficacy. More than 50% of the patient population receiving Epalrestat rated the treatment as an excellent treatment option in terms of efficacy and safety. Although Methylcobalamine also showed effectiveness and better tolerability, it was statistically lower than Epalrestat in patients' opinions. Very few adverse events were associated with both the drugs. The findings of the study ensure the effectiveness and safety of Epalrestat as a potential treatment option for diabetic neuropathy.

## Conclusion

Epalrestat, an aldose reductase inhibitor, seems to be a better alternative than Methylcobalamine in the treatment of diabetic neuropathy. In the present study, Epalrestat was clearly ahead of Methylcobalamine in efficacy parameters such as sensory loss, burning sensation, numbness, muscle cramps, and weakness. Epalrestat 50 mg thrice daily was better tolerated than Methylcobalamine 500 µg for a 12-week treatment period. Our study also confirms the safety of Epalrestat as there were no serious adverse events during the study. From the present study, we can conclude that Epalrestat has a better efficacy and safety profile than Methylcobalamine in the treatment of diabetic neuropathy.

## References

[1] Ide H, Fujiya S, Asanuma Y, Tsuji M, Sakai H, Agishi Y (1987). Clinical usefulness of intrathecal injection of Methylcobalamine in patients with diabetic neuropathy. Clin Ther.

[2] Yaqub BA, Siddique A, Sulimani R (1992). Effects of Methylcobalamine on diabetic neuropathy. Clin Neurol Neurosurg.

[3] Goto Y, Hotta N, Shigeta Y (1993). A placebo-controlled double-blind study of epalrestat (ONO-2235) in patients with diabetic neuropathy. Diabetes Med.

[4] Uchida K, Kigoshi T, Nakano S (1995). Effect of 24 weeks of treatment with epalrestat: An aldose reductase inhibitor on peripheral neuropathy in patients with non-insulin-dependent diabetes mellitus. Clin Ther.

[5] Hotta N, Sakamoto N, Shigeta Y, Kikkawa R, Goto Y (1996). Clinical investigation of epalrestat, an aldose reductase inhibitor, on diabetic neuropathy in Japan: Multicenter study. J Diabetes Compl.

[6] Ikeda T, Iwata K, Tanaka Y (1999). Long-term effect of epalrestat on cardiac autonomic neuropathy in subjects with non-insulin dependent diabetes mellitus. Diabetes Res Clin Pract.

[7] Iso K, Tada H, Kuboki K, Inokuchi T (2001). Long-term effect of epalrestat, an aldose reductase inhibitor, on the development of incipient diabetic nephropathy in type 2 diabetic patients. J Diabetes Compl.

[8] Diabetes Control and Complications Trial Research Group (1993). The effect of intensive treatment of diabetes on the development and progression of long-term complications in insulin-dependent diabetes mellitus. N Engl J Med.

[8a] Ohkubo Y, Kishikawa H, Araki E, Miyata T, Isami S, Motoyoshi S (1995). Intensive insulin therapy prevents the progression of diabetic microvascular complications in Japanese patients with non-insulin-dependent diabetes mellitus: A randomized prospective 6-year study. Diabetes Res Clin Pract.

[8b] UK Prospective Diabetes Study (UKPDS) Group (1998). Intensive blood-glucose control with sulphonylureas or insulin comparedd with conventional treatment and risk of complications in patients with type 2 diabetes (UKPDS 33). Lancet.

[9] Vinik AI, Mehrabyan A (2004). Diabetic neuropathies. Med Clin North Am.

[10] Hotta N (1997). New concepts and insights on pathogenesis and treatment of diabetic complications: Polyol pathway and its inhibition. Nagoya J Med Sci.

[11] Oates PJ, Mylari BL (1999). Aldose reductase inhibitors: Therapeutic implications for diabetic complications. Expert Opin Investig Drugs.

[12] Cameron NE, Eaton SE, Cotter MA, Tesfaye S (2001). Vascular factors and metabolic interactions in the pathogenesis of diabetic neuropathy. Diabetologia.

[13] Oates PJ (2002). Polyol pathway and diabetic peripheral neuropathy. Int Rev Neurobiol.

